# Plant terminators: the unsung heroes of gene expression

**DOI:** 10.1093/jxb/erac467

**Published:** 2022-12-08

**Authors:** Felipe F de Felippes, Peter M Waterhouse

**Affiliations:** Centre for Agriculture and the Bioeconomy, Institute for Future Environments, Queensland University of Technology (QUT), Brisbane, QLD, Australia; ARC Centre of Excellence for Plant Success in Nature & Agriculture, QUT, Brisbane, QLD, Australia; Centre for Agriculture and the Bioeconomy, Institute for Future Environments, Queensland University of Technology (QUT), Brisbane, QLD, Australia; ARC Centre of Excellence for Plant Success in Nature & Agriculture, QUT, Brisbane, QLD, Australia; University of Copenhagen, Denmark

**Keywords:** Gene expression, poly(A), polyadenylation, post-transcriptional gene silencing, silencing, small RNAs, terminator, transcription termination

## Abstract

To be properly expressed, genes need to be accompanied by a terminator, a region downstream of the coding sequence that contains the information necessary for the maturation of the mRNA 3ʹ end. The main event in this process is the addition of a poly(A) tail at the 3ʹ end of the new transcript, a critical step in mRNA biology that has important consequences for the expression of genes. Here, we review the mechanism leading to cleavage and polyadenylation of newly transcribed mRNAs and how this process can affect the final levels of gene expression. We give special attention to an aspect often overlooked, the effect that different terminators can have on the expression of genes. We also discuss some exciting findings connecting the choice of terminator to the biogenesis of small RNAs, which are a central part of one of the most important mechanisms of regulation of gene expression in plants.

## Teaser

The function of gene terminators goes beyond the formation of the mRNA 3ʹ end: they are a crucial element of gene expression and the production of regulatory small RNAs in plants.

## Introduction

Gene expression is a complex process vital for the proper development of plants and their adaptation to their surrounding environment. One key step in this process is the formation of the 3ʹ end of the newly synthesized mRNA molecule, which is mainly characterized by the cleavage and polyadenylation of the nascent transcript (Kumar *et al.*, 2019; Thore and Fribourg, 2019; Sun *et al.*, 2020). Pre-mRNA 3ʹ end processing requires the presence of the terminator, a genic element located downstream of the coding sequence that, once transcribed, is recognized by different protein complexes responsible for this step in mRNA biogenesis. Besides defining the mRNA posterior border, the addition of a poly(A) tail has important implications for the stability, maturation, nuclear export, and translation of the new transcript (Mandel *et al.*, 2008; Nicholson and Pasquinelli, 2019; Passmore and Coller, 2022). Processing of the mRNA 3ʹ end also plays a role in the termination of transcription, assisting with the release of RNA Polymerase II (Pol II) from the pre-mRNA template. There is, however, another facet involving this process and related factors that is less appreciated. In plants, the choice of the terminator and some sequences in the 3ʹ untranslated region (3ʹ UTR; the segment in the mRNA molecule after the stop codon, which includes part of the terminator sequence) can have a significant impact on the expression of transgenes, going beyond aspects usually associated with polyadenylation and transcription termination. The biogenesis of small RNAs (sRNAs) is one process that seems to be affected by different terminator sequences. These molecules have a critical role in the regulation of gene activity, a mechanism better known as silencing or RNA interference (RNAi). In this review, we will revisit the 3ʹ end maturation process, focusing on the impact that terminators can have on gene expression and the biogenesis of sRNAs.

## Pre-mRNA cleavage and polyadenylation

The formation of a mRNA 3ʹ extremity requires the activity of several protein complexes that recognize and bind to sequences in the pre-mRNA encoded by the terminator region. In mammals, where this mechanism is better characterized, the main complex involved in the processing of the pre-mRNA 3ʹ end is the Cleavage and Polyadenylation Speciﬁcity Factor (CPSF), which is further divided into two sub-complexes: the mammalian polyadenylation specificity factor (mPSF) and the mammalian cleavage factor (mCF). mPSF consists of CPSF160, CPSF30, WDR33, and Fip1, while three other proteins, CPSF73, CPSF100, and symplekin, form the mCF (Chan *et al.*, 2014; Schönemann *et al.*, 2014; Kumar *et al.*, 2019; Thore and Fribourg, 2019; Sun *et al.*, 2020). During pre-mRNA 3ʹ end processing, CPSF, via CPSF30 and WDR33, binds to the polyadenylation signal (PAS), a short sequence domain most often represented by the A(A/U)UAAA hexamer, while the endonuclease activity of CPSF73 is responsible for cutting the nascent transcript in a downstream position known as the cleavage site (Proudfoot and Brownlee, 1974, 1976; Beaudoing *et al.*, 2000; Tian *et al.*, 2005; Mandel *et al.*, 2006; Chan *et al.*, 2014; Schönemann *et al.*, 2014; Sun *et al.*, 2020). A stretch of adenine residues known as the poly(A) tail is then added to the 3ʹ end of the transcript from the cleavage site by the poly(A) polymerase (PAP) (Mandel *et al.*, 2008). Recruitment of PAP to the nascent transcript is done through interactions with mPSF subunit Fip1 (Kaufmann *et al.*, 2004). The remaining subunits, symplekin, CPSF100, and CPSF160, have important roles as scaffold proteins and in intra- and inter-complex interactions (Clerici *et al.*, 2018; Sun *et al.*, 2018, 2020; Zhang *et al.*, 2020).

Three other complexes participate in the maturation of the 3ʹ end of mRNAs in mammals, Cleavage Factor I and II (CFIm and CFIIm, respectively), and Cleavage Stimulation Factor (CstF). CFIm recognizes the upstream UGUA motif, while CstF and CFIIm bind, respectively, to U/GU- and G-rich sequences located after the cleavage site (Thore and Fribourg, 2019; Zhang *et al.*, 2020). Although CPSF can independently recognize the PAS (Chan *et al.*, 2014; Schönemann *et al.*, 2014), the efficiency and precision of the mRNA 3ʹ end formation are significantly influenced by CstF, CFIm, and CFIIm activity. Those complexes provide an additional hook onto the pre-mRNA, helping to position the 3ʹ end processing machinery and enhancing its activity (Takagaki *et al.*, 1990; Brown and Gilmartin, 2003; Venkataraman *et al.*, 2005; Grozdanov *et al.*, 2018; Schäfer *et al.*, 2018). CFIm also has a main role in the recognition and choice of alternative polyadenylation sites (Martin *et al.*, 2012; Zhu *et al.*, 2018), while CstF is known to bind to Pol II, being an important factor coupling polyadenylation and transcription termination (McCracken *et al.*, 1997; Fong and Bentley, 2001).

The process described above shows remarkable similarities with those of yeast, another system where mRNA cleavage and polyadenylation have been extensively studied, indicating that the mechanism of 3ʹ end formation is conserved across eukaryotes (Kumar *et al.*, 2019; Thore and Fribourg, 2019). This observation also extends to plants, where homologues for most mammalian and yeast subunits have been found, and their activities and protein–protein interactions have been validated (Yao *et al.*, 2002; Simpson *et al.*, 2003; Xu *et al.*, 2004; Delaney *et al.*, 2006; Forbes *et al.*, 2006; Herr *et al.*, 2006; Addepalli and Hunt, 2007; Hunt *et al.*, 2008, 2012; Zhao *et al.*, 2009; Yu *et al.*, 2019). Despite all these similarities, the processing of the mRNA 3ʹ end in plants also has its particularities. For instance, some protein interactions seem to be specific to plants, such as the one detected between CPSF100 and at least one of the PAPs (Elliott *et al.*, 2003). On the other hand, no interaction between CstF50 and CstF77 was detected in Arabidopsis, contrasting with what has been seen in mammals (Takagaki and Manley, 2000; Yao *et al.*, 2002). Interestingly, CstF50 seems to be absent in some species such as *Populus trichocarpa*, raising the possibility that this protein may be dispensable for the pre-mRNA cleavage and polyadenylation process in plants (Hunt *et al.*, 2012). Another important difference between plants and mammals (and also yeast) lies in the number of genes coding for each subunit. In plants, several of these proteins are formed by multigenic families and even one gene could have more than one isoform (Hunt, 2008; Hunt *et al.*, 2012). One possible consequence of this would be mRNA 3ʹ end machinery that may be a collection of distinct assemblies, each one containing proteins encoded by different members of the gene family, adding variability to the complex function. An example of this putative variability in function can be seen in CPSF30 and its larger, plant-specific isoform CPSF30L. While CPSF30 is most likely involved in the general formation of the 3ʹ end of transcripts, CPSF30L might be more specialized, acting on mRNAs containing *N*^6^-methyl-adenosine (m^6^A) modifications to prevent read-through transcription and the formation of chimeric mRNAs between neighbouring genes (Pontier *et al.*, 2019). But perhaps the most meaningful difference between the plant and mammalian system might be the architecture of their terminators and 3ʹUTR, which we will discuss in more detail in the next section. On that note, it is important to notice that despite the close association between polyadenylation and transcription termination, these are two distinct biochemical processes. In this review, the term ‘terminator’ will be used to indicate the genic region containing the PAS and other elements necessary for the polyadenylation and 3ʹ end processing of mRNAs. Nonetheless, the connections between transcription termination and polyadenylation will also be explored.

## Plant terminators

**Fig. 1.**
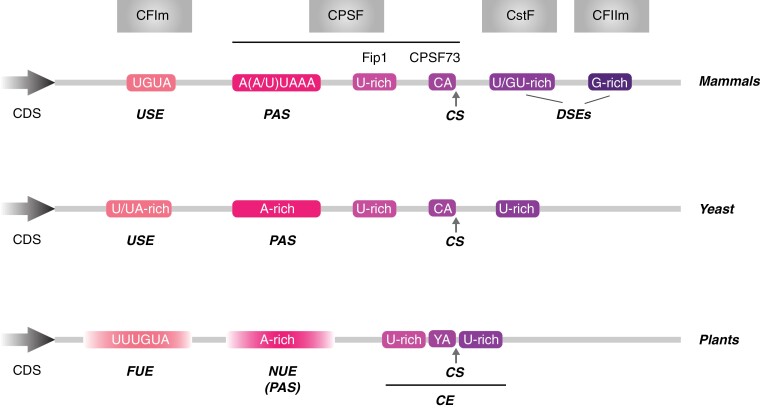
Schematic representation of the terminator regions of mammals, yeast, and plants. The *cis*-acting elements recognized by the 3’ end processing machinery are displayed (boxes of different colours on the terminator) and consensus sequences are given. The following elements are indicated: CDS, coding sequence; CE, cleavage element; CS, cleavage site; DSE, downstream element; FUE, far upstream element; NUE; near upstream element; PAS, polyadenylation signal; USE, upstream element. The borders of the plant FUE and NUE are diffused to highlight the variable size when compared with the other organisms. Protein complexes recognising the different cis-acting elements are shown on top of the figure.

The most prominent feature of mammalian terminators is the PAS, characterized by the consensus AAUAAA motif. This sequence and a close variant (AUUAAA) are present in approximately 60% and 15% of the mammalian pre-mRNAs, respectively. The PAS is located about 20 nt upstream of the cleavage site, which usually comes after a CA dinucleotide. Three other sequences are frequently found in mammalian terminators: the UGUA motif recognized by CFIm (also known as upstream element—USE, due to its location before the cleavage site) and a G/U- and a G-rich region, both located after the cleavage site (and therefore referred to as downstream elements—DSE) and bound by the CstF and CFIIm complexes, respectively (Fig. 1) (Kumar *et al.*, 2019; Thore and Fribourg, 2019; Sun *et al.*, 2020).

Early studies led to a tripartite model suggesting the presence of three main *cis*-elements in the terminator of plants: the far upstream element (FUE), the near upstream element (NUE), and the cleavage site (CS) (Hunt, 1994, 2008; Rothnie, 1996). The latter was later expanded to include two short U-rich regions located immediately up- and downstream of the cleavage site and referred to as the cleavage element (CE) (Loke *et al.*, 2005). This alternative configuration of the plant regulatory unit shows significant differences from its mammalian counterpart. The FUE is a U-rich region that can span 60–130 nt and is located 30 nt or more upstream of the cleavage site (Hunt, 2008; Bernardes and Menossi, 2020). This element is involved with the efficiency of mRNA 3ʹ end processing and shows some resemblance with the mammalian USE (Hunt, 1994; Rothnie, 1996). Indeed, the sequence UUUGUA, a recurrent motif in FUEs, is similar to the region bound by the mammalian CFIm complex. Moreover, the UUUGUA motif has been shown to induce the processing of cryptic poly(A) sites if introduced near its PAS (Sanfacon *et al.*, 1991; Rothnie *et al.*, 1994). This feature might be similar to the mechanism involving CFIm and the choice of alternative polyadenylation sites (Martin *et al.*, 2012; Zhu *et al.*, 2018). The NUE is the plant terminator element containing the polyadenylation signal. It is located 13–30 nt upstream of the cleavage signal and is characterized by being A-rich (Hunt, 2008; Bernardes and Menossi, 2020). The conserved mammalian AAUAAA motif is also the preferred sequence found in the plant’s PAS; however, it is only associated with about 10% of the plant poly(A) sites (Loke *et al.*, 2005; Shen *et al.*, 2008; Sherstnev *et al.*, 2012). In addition, the plant PAS can exceed 6 nt and tolerates more variations than the mammalian system (Hunt, 1994; Rothnie, 1996). The third element, CE, consists of the cleavage site itself embedded within a U-rich region (Loke *et al.*, 2005). The tendency in eukaryotes is for the most common dinucleotide immediately before the cleavage site to be either UA or CA (YA) (Graber *et al.*, 1999; Loke *et al.*, 2005). These *cis*-elements can be found more than once in a plant terminator, with each poly(A) site requiring its own NUE, but with FUEs potentially regulating more than one NUE (Hunt, 2008). This characteristic of plant terminators might explain the extreme heterogeneity of mRNA 3ʹ ends in plants (Sherstnev *et al.*, 2012). Nonetheless, despite all the differences from other systems and the lack of sequence conservation within *cis*-elements, it is possible to identify a clear pattern of alternating U- and A-rich regions in the plant terminator that resembles those found in other organisms, such as mammals and especially yeast, indicating that the terminator architecture across kingdoms might be more similar than previously believed (Fig. 1) (Loke *et al.*, 2005; Sherstnev *et al.*, 2012; Kumar *et al.*, 2019).

## Terminators, polyadenylation and regulation of gene expression

### The canonical role of 3ʹ end formation in gene expression

The impact of polyadenylation on the mRNA is tremendous: it constitutes an important regulatory mechanism affecting gene expression at different levels. The poly(A) tail plays a central role in the export of mRNAs from the nucleus to the cytoplasm. Unadenylated mRNAs tend to accumulate in the nucleus, leading to a decrease in protein accumulation, a scenario that can be reverted by the presence of a synthetic poly(A) tail (Eckner *et al.*, 1991; Huang and Carmichael, 1996; Dower *et al.*, 2004). The poly(A) is also crucial to the stability of the mRNA. Despite reports showing that several well-expressed genes have mRNAs with short poly(A) tails (Choi and Hagedorn, 2003; Meijer *et al.*, 2007), it is generally accepted that transcripts with longer poly(A) are better protected from decay and degradation, and, as a consequence, have higher expression levels (Mandel *et al.*, 2008; Nicholson and Pasquinelli, 2019; Bernardes and Menossi, 2020; Passmore and Coller, 2022). In eukaryotes, the bulk of mRNA decay requires the action of XRN1 (or its plant homologue XRN4), an exoribonuclease that degrades RNA in the 5’→3’ direction and a large complex of 3’→5’ exonucleases known as the exosome. mRNA decay requires first the shortening of the poly(A) tail, followed by 5’ cap removal before degradation by XRN1 and the exosome (Passmore and Coller, 2022). The interaction of the poly(A) with the cytoplasmatic poly(A)-binding protein (PABPC) is key for the protection offered by the poly(A) tail. The binding of PABPC to poly(A) seems to protect the 3’ end of the mRNA from exonucleases (Mandel *et al.*, 2008; Nicholson and Pasquinelli, 2019; Passmore and Coller, 2022). In accordance, sequestering PABPC in experiments using *in vitro* degradation systems leads to the destabilization of reporter RNAs, while adding excess amounts of this binding protein can inhibit deadenylation (Bernstein *et al.*, 1989; Wormington *et al.*, 1996; Ford *et al.*, 1997; Tucker *et al.*, 2002; Viswanathan *et al.*, 2004). The poly(A) tail is also an important factor stimulating mRNA translation, with several reports correlating polyadenylation with enhanced translational rates (Passmore and Coller, 2022). The poly(A) tail acts synergistically with the 5’ cap to regulate the efficiency of translation (Gallie, 1991; Preiss *et al.*, 1998), a process that again has the PABPC–poly(A) interaction in a pivotal position. The translation initiation process starts with eIF4E binding to the mRNA 5’ cap, which is followed by the recruitment of two other translation initiation factors, eIF4G and eIF4A. The formation of this complex is required for recruiting the 40S small ribosomal subunit, thus loading the ribosome onto the mRNA (Farache *et al.*, 2022). PABPC interacts with eIF4G (Tarun and Sachs, 1996), an association that stabilizes the interaction of eIF4E with the 5’ cap (Borman *et al.*, 2000). Moreover, PABPC increases the ATPase and the RNA helicase activity of eIF4A resulting in increased rates of translation (Bi and Goss, 2000).

In addition to its contribution to the stability and translational status of mRNAs, processing of the 3’ end of transcripts is essential at the end of the transcriptional process (Zaret and Sherman, 1982; Whitelaw and Proudfoot, 1986; Logan *et al.*, 1987). Proper termination is important to ensure that mRNAs are fully transcribed and that transcription does not continue indefinitely, which could result in the generation of aberrant transcripts, interference with the transcription of neighbouring genes, sRNA generation, and even triggering of DNA damage and genome instability due to disruption of DNA replication (Proudfoot, 2016). Variations in the termination position can also lead to mRNAs with diverse 3’ UTRs with different regulatory properties. For instance, longer 3’ UTRs could contain alternative polyadenylation sites, which could have important consequences for the regulation of gene expression (Tian and Manley, 2017). Long 3’ UTRs have also been shown to be a trigger of nonsense-mediated mRNA decay (Kertész *et al.*, 2006). For termination to happen, it is necessary that Pol II is released from its template. Two models are predominantly used to explain how this process happens in eukaryotes, and both are dependent on the events leading to mRNA polyadenylation. The first one, referred to as the allosteric model, proposes that Pol II, upon encountering a functional PAS, undergoes conformational changes resulting in the polymerase slowing down and its gradual release from the DNA template (Porrua *et al.*, 2016; Proudfoot, 2016). An important factor in this mechanism is the CFIIm protein Pcf11 (and its orthologues in other species), which causes Pol II to dissociate from the nascent transcript (Zhang *et al.*, 2005; Zhang and Gilmour, 2006). The effect of Pcf11 on Pol II is through its interaction with the C-terminal domain (CTD) of the largest Pol II subunit, an important region coupling several different processes associated with transcription. Pfc11 forms a bridge between the CTD and the RNA, an interaction that is believed to cause conformational changes to Pol II and help to dismantle the elongation complex (Gilmour and Fan, 2008; Mandel *et al.*, 2008). Changes in Pol II processivity also involve components of CPSF and CstF complexes, such as CPSF73 and CstF64, whose absence results in a reduction of the polymerase pausing activity (Nojima *et al.*, 2015). In addition, two plant-specific proteins, FPA and the CPSF30 larger isoform (CPSF30L), also seem to play a role in assisting transcriptional termination. Plants carrying mutations affecting the activity of either one of these proteins show increased levels of transcriptional read-through that can result in the formation of chimeric mRNAs between neighbouring genes (Duc *et al.*, 2013; Pontier *et al.*, 2019). After cleavage by CPSF73, the fragment of the nascent transcript that is still attached to the elongation complex is degraded by a 5’→3’ exonuclease, which in mammals is XRN2. A kinetic competition exists in which XRN2 pursues the elongating polymerase, eventually reaching Pol II and eliciting termination. Due to the similarities to a naval battle, this mechanism is known as the torpedo model of transcription termination (Porrua *et al.*, 2016; Proudfoot, 2016). A unified allosteric–torpedo model also exists, in which the chase of Pol II by the exonuclease is facilitated by a pausing effect triggered by the polymerase interaction with PAS and the polyadenylation machinery (Luo *et al.*, 2006; Fong *et al.*, 2015; H. Zhang *et al.*, 2015; Eaton *et al.*, 2020).

### Promoter-proximal termination and the control of gene expression

During transcription, Pol II elongation rates throughout the gene body are not uniform, varying within and between genes. This variation in the pace at which polymerization occurs can have important consequences for co-transcriptional processes and gene expression (Jonkers and Lis, 2015). For instance, mutations affecting Pol II catalytic activity in yeast revealed a connection between the polymerization speed and the selection of transcription start sites (TSSs) (Kaplan *et al.*, 2012). In plants, Pol II mutations predicted to decelerate the polymerase activity are not viable, while modifications with an opposite effect result in strong developmental defects associated with changes in splicing efficiency and increased read-through transcription (Leng *et al.*, 2020). There are at least two moments during transcription when Pol II shows a dramatic change in elongation rates. This is readily visible by analysing the profile of Pol II transcription across eukaryotic genes, with Pol II accumulating at two different regions of the gene, one just downstream of the TSS and the second near the PAS (Jonkers and Lis, 2015). As discussed above, the accumulation peak at the end of the gene reflects a reduction in the Pol II movement linked to the termination of transcription and polyadenylation (Porrua *et al.*, 2016; Proudfoot, 2016). The meaning of the Pol II accumulation near the TSS, on the other hand, is still debatable. It is usually believed that such enrichment near the TSS is caused by a stable pausing of engaged Pol II. This pausing in the polymerase activity could be an important window of opportunity for the regulation and coordination of transcription elongation with other processes. In addition, Pol II pausing near the TSS supports active transcription by maintaining an open chromatin state near promoters, and it is also an important mechanism allowing for a quick transcriptional response from stimulus-controlled pathways (Jonkers and Lis, 2015; Mayer *et al.*, 2017). Alternatively, the accumulation of the polymerase near the TSS could also be interpreted as a consequence of premature transcription termination (PPT) followed by Pol II turnover (Kamieniarz-Gdula and Proudfoot, 2019). Promoter-proximal PPT could indeed be a significant mechanism influencing the Pol II distribution profile near the TSS, especially in organisms (such as plants) that do not have counterparts for components of the negative elongation factor complex (NELF), which in metazoans plays an important part in Pol II pausing (Hajheidari *et al.*, 2013; Jonkers and Lis, 2015; Mayer *et al.*, 2017; Kamieniarz-Gdula and Proudfoot, 2019). The role of transcriptional termination in Pol II accumulation near the TSS is further supported by the identification in plants of short promoter-proximal RNAs (sppRNAs) (Thomas *et al.*, 2020). These molecules, which are capped and polyadenylated, are the product of transcription initiating at the canonical TSS (near the promoter) followed by promoter-proximal PPT, resulting in transcripts for which location correlates with the peak accumulation of Pol II near the gene 5’ end. Thus, sppRNA features are compatible with the scenario in which Pol II accumulation near the TSS is a result of the polymerase failing to enter the productive elongation phase due to premature termination. More importantly, promoter-proximal termination and its effect on Pol II activity offer yet another example of the importance of transcriptional termination for the regulation of gene expression, and it also expands the definition of terminators in plants.

### Terminator choice and its consequences for transgene expression

Despite the impact transcript 3’ end formation can have on different aspects of mRNA life, the importance of terminators for the expression of transgenes does not seem to receive the same attention as other regulatory regions such as promoters. This becomes evident when we look at several collections of expression vectors used in plants. While alternatives to the widely used cauliflower mosaic virus (CaMV) 35S promoter are frequently available and plasmid versions for promoter customization usually exist, the same cannot be said of the terminator (Karimi *et al.*, 2005; Earley *et al.*, 2006; Coutu *et al.*, 2007; Nakagawa *et al.*, 2007; Rybel *et al.*, 2011). A likely explanation for this would be a misperception that the sole function of the terminator is to trigger polyadenylation, and as long as a gene is supported by a functional terminator, its expression would not be affected by the deleterious effects associated with the lack of a poly(A) tail. There are, however, an ever-growing number of studies showing that the choice of terminator can have a crucial impact on the final levels of transgene expression.

One of the first pieces of evidence showing the effects distinct terminators can have on gene expression came from the study of two RuBisCo genes from petunia, *SSU301* and *SSU911*. Compared with the former, *SSU911* expression can be up to 100-fold lower. By using chimeric constructs in which sequences of both genes were exchanged at different positions, Dean *et al.* (1989) were able to dramatically increase the expression of the weaker *SSU911* using only the region located downstream of the *SSU301* coding sequence. In the same year, another study tested the effect of different plant terminators on the expression of a reporter gene. Although expression levels were not so different in a transient system, a sharp contrast was observed for stable transgenes, with expression varying up to 60-fold among various constructs using distinct terminatory sequences (Ingelbrecht *et al.*, 1989). A similar observation regarding the variation in gene expression caused by the choice of terminator has been made over the years, with different regulatory elements and expression systems tested (Marshall *et al.*, 1997; Chen *et al.*, 1998; Richter *et al.*, 2000; Ali and Taylor, 2001a; Schünmann *et al.*, 2003; Yang *et al.*, 2009; Nagaya *et al.*, 2009; Hiwasa-Tanase *et al.*, 2011; Schaart *et al.*, 2011; Li *et al.*, 2012; Limkul *et al.*, 2015; Rosenthal *et al.*, 2018; Pérez-González and Caro, 2018; Diamos and Mason, 2018; Wang *et al.*, 2020; de Felippes *et al.*, 2020). In some of these cases, the selection of a terminator was the most significant factor, among different genic elements tested, affecting gene expression levels (Richter *et al.*, 2000; de Felippes *et al.*, 2020). Curiously, the most popular terminators used for gene expression in plants, CaMV 35S, *nopaline synthase* (*nos*), and *octopine synthase* (*ocs*; these last two from *Agrobacterium tumefaciens*), are far from being the most efficient. Several studies reported an improvement in transgene expression levels when different 3’ regulatory regions, other than the three mentioned above, are used (reviewed by Bernardes and Menossi, 2020). For some terminators, such as the *Flaveria bidentis Me1* (NADP-malic enzyme), a staggering 440-fold difference in expression efficiency was detected (Marshall *et al.*, 1997).

In most cases, the features of one terminator seem to be interspecific, even between monocots and dicots. For instance, the Arabidopsis-derived *HEAT SHOCK PROTEIN 18.2* (*HSP18.2*) terminator showed comparable superior efficiency when used to drive transgene expression in its original organism or other plants such as tomato, rice, lettuce, or *Nicotiana benthamiana* (Nagaya *et al.*, 2009; Matsui *et al.*, 2011; Hirai *et al.*, 2011; Kurokawa *et al.*, 2013; Pérez-González and Caro, 2018; Diamos and Mason, 2018; de Felippes *et al.*, 2020). However, there are also cases of terminators displaying characteristics that are specific to certain species. The 3’ regulatory region of the Rubisco small subunit (*RBCS1A*) from Arabidopsis can support satisfactory gene expression when used for the generation of transgenic Arabidopsis plants. In contrast, its efficiency is rather poor in *N. benthamiana* (at least in transient expression assays), where its use results in very low levels of gene expression and it is also a significant factor triggering transgene silencing (de Felippes *et al.*, 2020). Activity of some terminators also seems to vary even within organisms, in a tissue-specific matter. The use of the *GLUTELIN B-1* (*Glub-1*) terminator from rice was shown to result in more transgene accumulation in seeds when compared with the *nos* regulatory unit. However, this advantage was not observed in leaves, where both terminators delivered a similar output (Yang *et al.*, 2009). While these examples might be simple exceptions, they also point to an interesting possible new layer of gene regulation that is usually associated with the activity of promoters.

Another unique feature of terminators that can have a significant impact on gene expression was observed when two different 3’ regulatory units were combined. Transgene expression in Arabidopsis, sugarcane, sorghum, and tobacco was dramatically improved when the 35S and the *nos* terminators were coupled together as a double terminator in the expression cassette compared with constructs having either one of them on its own (Luo and Chen, 2007; Beyene *et al.*, 2011). Similarly, among several arrangements tested, the combination of the *HSP* with the tobacco *Extensin* (*Ext*) terminator was found to be the most efficient set-up for the transient expression of proteins in *N. benthamiana* leaves, providing higher yields than constructs using these terminators in a single configuration. Moreover, the *HSP*–*Ext* arrangement showed better results than having the same terminator duplicated (*HSP*–*HSP*) and it was also more efficient than the combination 35S–*nos* (Yamamoto *et al.*, 2018). Interestingly, the context in which double terminators are arranged in the expression cassette also seems to make a difference to the efficiency of gene expression. While the 35S terminator seems to perform better when placed 5’ of the *nos* terminator in a double regulatory unit having both elements, the opposite happens when it is paired with the *Ext* terminator (Diamos and Mason, 2018). Most likely, sequences in the terminator placed upstream can perturb the activity of the regulatory unit located in the second position. For instance, positioning the 35S terminator directly upstream of the *nos* terminator led to the activation of a cryptic PAS changing the dominant polyadenylation site in the latter (Sanfacon *et al.*, 1991).

But how exactly does the choice of terminator have an impact on gene expression? From the studies investigating the impact of different plant terminators on transgene expression in which molecular analyses were conducted, the most common observation was a positive correlation between gene expression and mRNA steady-state levels (Dean *et al.*, 1989; Ingelbrecht *et al.*, 1989; Chen *et al.*, 1998; Richter *et al.*, 2000; Ali and Taylor, 2001b; Yang *et al.*, 2009; Nagaya *et al.*, 2009; Hiwasa-Tanase *et al.*, 2011; Schaart *et al.*, 2011; Hirai *et al.*, 2011; Li *et al.*, 2012; Kurokawa *et al.*, 2013; Rosenthal *et al.*, 2018; Wang *et al.*, 2020; de Felippes *et al.*, 2020). This indicates, at least for those cases, that the effect of the terminator choice on gene expression occurs at the level of transcription and/or mRNA stability, rather than translation. In line with this, the efficiency difference between certain terminators has been linked with their ability to trigger transcription termination, as shown by an increase in read-through transcription on genes under the control of the weaker terminator (Hiwasa-Tanase *et al.*, 2011; Rosenthal *et al.*, 2018; de Felippes *et al.*, 2020). The precision in which transcripts are cleaved for the addition of the poly(A) tail is also a factor identified to explain differences in terminator strength. Mapping of the polyadenylation site in transcripts controlled by the *nos* terminator shows a diffuse pattern, with cleavage happening in several different locations after the PAS. In contrast, in the case of the *HSP*, *Glub-1* and *Miraculin* (*Mir*) 3’ regulatory regions, all of which support stronger transgene expression than the *nos* terminator, polyadenylation occurs mainly at one specific site (Yang *et al.*, 2009; Hiwasa-Tanase *et al.*, 2011; de Felippes *et al.*, 2020). Moreover, in tissues where the *Glub-1* terminator does not overperform the *nos*, poly(A) sites are also diffused, resembling what is seen for the bacterial regulatory region and further associating the polyadenylation precision with the terminator efficiency (Yang *et al.*, 2009). More recently, it has been reported that terminators leading to higher transgene expression levels are also more effective in protecting transgenes from transcriptional gene silencing (TGS) and post-transcriptional gene silencing (PTGS) (Pérez-González and Caro, 2018; de Felippes *et al.*, 2020, 2022). This aspect of the terminator function, which is linked to the presence of sRNAs, is the theme of our next section and will be discussed in more detail there. In summary, the mechanisms underlying the difference in terminators’ efficiency are still poorly characterized and are most likely multi-factorial and dependent on the choice of the terminator. The variability found in the sequence and arrangement of the different elements in a plant terminator most likely plays a major role, impacting how the processes linked to the mRNA 3’ end formation unfurl, and consequently affecting several aspects of mRNA biology. This is well illustrated by experiments showing that the distance of the mammalian upstream element UGUA to the poly(A) signal is an important element for the efficiency of transcript cleavage (Zhu *et al.*, 2018).

### Terminators and sRNA-mediated silencing

Gene silencing mediated by sRNAs is one of the most important mechanisms of regulation of gene expression in plants: it is essential for proper plant development and reproduction and for adaptation to stress, and it is a central component of the defence mechanism against viruses (Li *et al.*, 2017). The main effector protein in the gene silencing pathway is ARGONAUTE (AGO), which forms, together with the 20–24 nt sRNA molecule, the core of what is known as the RNA-induced silencing complex (RISC). The sRNA’s role is to confer sequence specificity to the RISC complex, allowing AGO to interact with targets that share sequence complementarity with the sRNA. RISC can act transcriptionally (TGS) by inducing DNA methylation or post-transcriptionally (PTGS) by inhibiting translation or promoting mRNA cleavage, which is the most common outcome in plants. In all these cases, sRNA action can result in a decrease or even complete knockout of gene expression (Carthew and Sontheimer, 2009; Bologna and Voinnet, 2014).

Transgenes are particularly prone to sRNA-mediated silencing, with terminators and the process of mRNA 3’ end formation playing a key role in this (de Felippes and Waterhouse, 2020). In two independent mutagenesis screens designed to investigate this susceptibility of transgenes to silencing, almost all genes identified were directly involved with polyadenylation and transcription termination (Herr *et al.*, 2006; de Felippes *et al.*, 2020). In line with this observation, transcripts expressed from cassettes lacking a terminator were strong sources of sRNAs and became silenced, while the presence of double terminators had the opposite effect, inhibiting transgene-derived sRNA production and improving expression (Luo and Chen, 2007; Nicholson and Srivastava, 2009). Compared with a classic transgene expression cassette consisting of an intronless reporter gene under the control of the CaMV 35S promoter and terminator, expression of a green fluorescent protein reporter containing introns and the Rubisco regulatory sequences resulted in less sRNA production and did not show signs of systemic silencing (Dadami *et al.*, 2013, 2014). Later, it was shown that out of these three genetic elements, the terminator was the one that conferred the highest protection against silencing and contributed the most to the final levels of gene expression (de Felippes *et al.*, 2020). Similarly, another study reported that different terminators can have distinct effects on the methylation of promoter regions, which is one of the outcomes of TGS (Pérez-González and Caro, 2018).

The mechanisms leading to the terminator-dependent generation of sRNAs are still not completely understood. The precursors of sRNAs are long double-stranded RNAs that are recognized by DICER-LIKE (DCL) enzymes and processed into molecules of 20–24 nt in size (Carthew and Sontheimer, 2009; Bologna and Voinnet, 2014). There are different sources of dsRNA in the plant cell, one of them being through the action of the RNA-Dependent RNA Polymerase 6 (RDR6), which converts single-stranded RNA into dsRNA (Willmann *et al.*, 2011). The recruitment of RDR6 to its template can happen through different mechanisms, one of them based on the presence of aberrant transcripts, such as the ones originating from a weak mRNA termination (for detailed reviews on the different mechanisms leading to RDR6-dependent sRNA generation, refer to de Felippes, 2019; de Felippes and Waterhouse, 2020; Hung and Slotkin, 2021). Luo and Chen (2007) demonstrated that sRNA production from transcripts expressed without a terminator was dependent on RDR6 activity. More importantly, these transcripts were the result of read-through transcription and lacked a poly(A) tail. The authors also showed that unpolyadenylated mRNAs resulting from abortive transcription elongation and premature termination were also targeted by RDR6 for sRNA generation. Indeed, it was later confirmed that the lack of the poly(A) was a central feature leading RDR6 to select aberrant poly(A)-less mRNAs over canonical polyadenylated transcripts as templates (Baeg *et al.*, 2017). Removal of the poly(A) was also identified as a factor enhancing dsRNA synthesis by RDR6 *in vitro* (Sakurai *et al.*, 2021). The effect different terminators have on the generation of sRNAs from transcripts they regulate seems to be largely dependent on their efficiency in preventing read-through transcription. In *N. benthamiana* transient assays, the high levels of sRNAs generated from transcripts driven by the Arabidopsis *RBCS1A* terminator were linked to an elevated degree of read-through transcription, especially when compared with the other terminators tested and for which the presence of sRNAs was not as abundant (de Felippes *et al.*, 2020). More recently, the characterization of the *HSP* terminator from Arabidopsis led to the identification of a sequence domain that seems to be one of the main reasons for the high efficiency of this regulatory region in protecting transcripts from spawning sRNAs. This newly identified region is crucial to keep read-through transcription to a minimum, and consequently keep transcripts under the control of the *HSP* terminator away from sRNA generation. Interestingly, the protective features of this region can be transferred to ‘weaker’ terminators, leading to increased transgene expression due to a reduction in read-through transcription and sRNA accumulation (de Felippes *et al.*, 2022). The importance of read-through transcription in the biogenesis of sRNAs is corroborated by the molecular analysis of transcription termination and polyadenylation mutants. In these lines, an increase in read-through transcription was evident, and it was also associated with the higher levels of sRNAs in these plants (Herr *et al.*, 2006; de Felippes *et al.*, 2020; Krzyszton and Kufel, 2022).

**Fig. 2.**
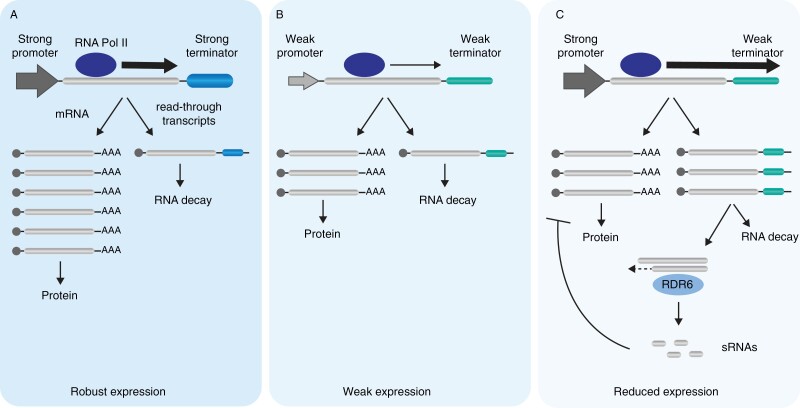
Gene harmony model. For most genes, the promoter and terminator strengths are balanced (A, B), resulting in gene expression levels that are mainly dependent on the promoter activity. Eventual read-through transcripts are efficiently processed by the RNA decay machinery, and hence no sRNA production occurs. In cases in which the promoter and terminator efficiency is unbalanced (as for many transgenic constructs) (C), read-through transcription surpasses the capacity of the RNA decay machinery and aberrant transcripts become templates for dsRNA synthesis by RDR6. As a result, sRNAs are generated leading to silencing of the gene of origin.

While the importance of terminators and the process of mRNA 3’ end formation in sRNA-dependent silencing of transgenes is becoming increasingly evident, their role in the biogenesis of sRNAs from endogenes is less clear. With the exception of a few specialized cases, such as the biogenesis of phased small interfering RNAs (phasiRNAs), endogenes do not seem to be natural templates for the RDR6-dependent production of sRNAs. Instead, aberrant transcripts from endogenes are mostly degraded by the RNA decay pathway, consisting of XRN4 and the RNA exosome (Souret *et al.*, 2004; Moreno *et al.*, 2013; X. Zhang *et al.*, 2015; Yu *et al.*, 2015; Branscheid *et al.*, 2015). However, mutations suppressing the activity of this degradation pathway result in increased levels of sRNAs originating from endogenes, indicating a competition between the RNA decay and the silencing pathways (Gregory *et al.*, 2008; X. Zhang *et al.*, 2015; Yu *et al.*, 2015; Branscheid *et al.*, 2015). This competition also seems to exist for transgenes, despite their already discussed susceptibility to silencing. In XRN4 and exosome knockout lines, transgene silencing is enhanced (Gazzani *et al.*, 2004; Moreno *et al.*, 2013; X. Zhang *et al.*, 2015; Yu *et al.*, 2015; Branscheid *et al.*, 2015). A few factors can explain the apparent dominance of the RNA decay machinery over RDR6 and the silencing pathway in the case of endogenes. Likely, endogenous loci giving origin to sRNAs due to poor transcription termination were negatively selected during evolution. As a consequence, genes evolved a balance between transcriptions and termination, reducing the levels of read-through transcription to a minimum. Eventually, aberrant mRNAs resulting from poor termination would occur at levels that can be efficiently processed by the RNA decay pathway. We refer to this as gene harmony. In contrast, most transgenes do not show this ‘harmony’ and would be unbalanced regarding the strength of their promoters and terminators. Indeed, the use of strong constitutive promoters such as the CaMV 35S is usually cited as one of the reasons for the susceptibility of transgenes to silencing (de Felippes and Waterhouse, 2020). Matching these strong promoters with more efficient terminators would restore the balance between these two regulatory regions, decreasing read-through transcription and bringing aberrant transcripts to a lower level at which they can be entirely processed by the RNA decay machinery, thus avoiding RDR6 and silencing. The gene harmony model is illustrated in Fig. 2. Still, there could be situations in which the balance between endogenous promoter and terminator is broken, leading to unusually high levels of aberrant transcript and ultimately silencing. This would be particularly relevant in the case of inducible and tissue-specific promoters. Biotic stresses can also lead to changes in the kinetics of the termination process. In mouse fibroblast cells, heat shock, osmotic, and oxidative stresses were shown to increase read-through transcription (Vilborg *et al.*, 2017). On the other hand, colder temperatures seem to have the opposite effect on plants, with read-through transcription being reduced after the stress is applied (Kindgren *et al.*, 2019). The consequences of these changes for the production of sRNAs from endogenes are not clear and will have to be further investigated.

## Final remarks

In conclusion, the process of transcript termination is strongly conserved across eukaryotes but with some diversity and kingdom-specific characteristics; in plants terminators appear to have fewer rigorous constraints in their motifs than in animals. The importance of terminators in the expression of both transgenes and endogenes has not been well recognized in the past but recent insights, such as the role of sRNAs induced by readthrough transcription, may well herald a greater appreciation of terminator diversity.
